# Subcellular targeting and dynamic regulation of PTEN: implications for neuronal cells and neurological disorders

**DOI:** 10.3389/fnmol.2014.00023

**Published:** 2014-04-01

**Authors:** Patricia Kreis, George Leondaritis, Ivo Lieberam, Britta J. Eickholt

**Affiliations:** ^1^MRC Centre for Developmental Neurobiology, King's College LondonLondon, UK; ^2^Institute of Biochemistry, Charité – Universitätsmedizin BerlinBerlin, Germany

**Keywords:** PTEN phosphohydrolase, neuronal morphology, synaptic transmission, membranes, PI3K/AKT/mTOR

## Abstract

PTEN is a lipid and protein phosphatase that regulates a diverse range of cellular mechanisms. PTEN is mainly present in the cytosol and transiently associates with the plasma membrane to dephosphorylate PI(3,4,5)P3, thereby antagonizing the PI3-Kinase signaling pathway. Recently, PTEN has been shown to associate also with organelles such as the endoplasmic reticulum (ER), the mitochondria, or the nucleus, and to be secreted outside of the cell. In addition, PTEN dynamically localizes to specialized sub-cellular compartments such as the neuronal growth cone or dendritic spines. The diverse localizations of PTEN imply a tight temporal and spatial regulation, orchestrated by mechanisms such as posttranslational modifications, formation of distinct protein–protein interactions, or the activation/recruitment of PTEN downstream of external cues. The regulation of PTEN function is thus not only important at the enzymatic activity level, but is also associated to its spatial distribution. In this review we will summarize (i) recent findings that highlight mechanisms controlling PTEN movement and sub-cellular localization, and (ii) current understanding of how PTEN localization is achieved by mechanisms controlling posttranslational modification, by association with binding partners and by PTEN structural or activity requirements. Finally, we will discuss the possible roles of compartmentalized PTEN in developing and mature neurons in health and disease.

## Introduction

Phosphatase and tensin homolog located on chromosome 10 (PTEN) was originally characterized as a tumor suppressor that can inhibit proliferation, migration, cell growth, and apoptosis in a number of different cells. Subsequently it became apparent that, in addition to its role as a tumor suppressor, PTEN has many roles in the central nervous system (CNS) during the different stages of brain development and in adulthood. PTEN is highly expressed in neurons (Lachyankar et al., 2000; Chadborn et al., 2006) and recent work indicates that de-regulation of PTEN affects important neuronal functions in the nervous system, which have been attributed to its role in controlling neurogenesis, neurite outgrowth, synaptogenesis, and synaptic plasticity (Van Diepen and Eickholt, 2008; Zhou and Parada, 2012). Human germline PTEN mutations or conditional deletions of PTEN in mice have provided insights into possible causes associated with neurological disorders such as macrocephaly, ataxia, seizures, mental retardation, and autism (Backman et al., 2001; Kwon et al., 2006; Van Diepen and Eickholt, 2008; Zhou and Parada, 2012). Also, inhibition of PTEN activity is currently seen as a persuasive target for increasing regenerative capacities of neurons affected in degenerative conditions, or following injury to the nervous system (Park et al., 2008).

### PTEN, a phosphoinositide 3-phosphatase that regulates PI3K (phosphoinositide 3 kinase) signaling

**Figure 1:**
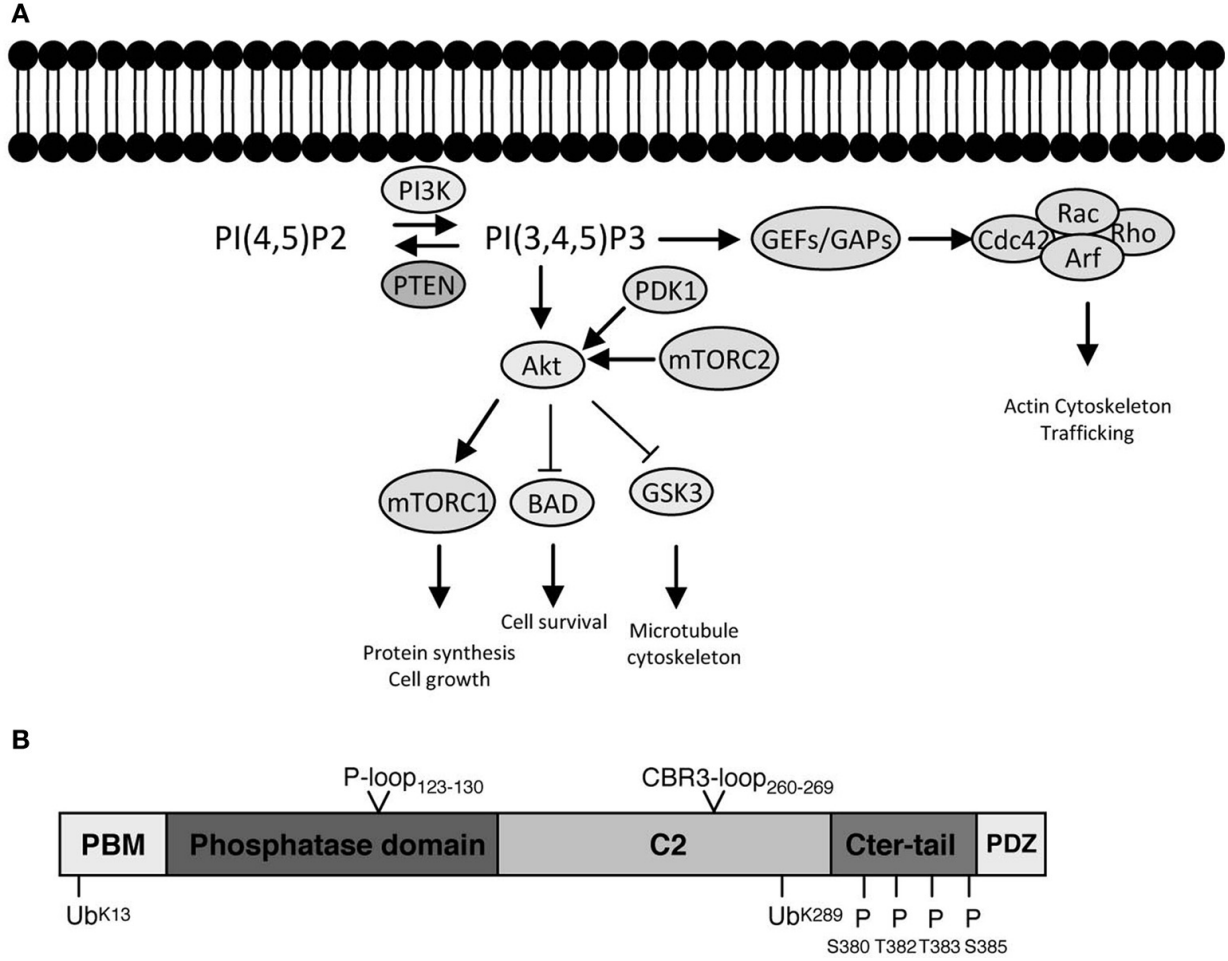
**PI3K signaling, PTEN, and downstream effector pathways. (A)** One of the most prominent PIP3 effectors is the protein kinase Akt. Upon PI3K activation, Akt translocates from the cytosol to the membrane, where it binds PIP3 and is phosphorylated and activated by PDK1 (phosphoinositide-dependent kinase 1) and the rapamycin-insensitive complex containing mTOR (mammalian target of rapamycin), mTORC2. Akt is considered as a master regulator of cell growth and proliferation, as well as survival, by phosphorylating and regulating the activity of several proteins, which convey growth-factor-derived signals to cellular functions. For example, Akt phosphorylates and inactivates TSC2 (Tuberous Sclerosis Complex 2), which, in a complex with TSC1, is responsible for inhibition of RHEB (Ras Homolog Enriched in Brain). Thus, release of this inhibition results in activation of mTORC1 complex, which is important for promoting protein translation and accelerating cell growth in neurons. Its deregulation is associated with several neurodevelopmental disorders (Costa-Mattioli and Monteggia, 2013). Akt also phosphorylates and inactivates GSK3 (glycogen synthase kinase 3), a serine/threonine kinase that has been associated with a number of neuronal responses, particularly during neuronal development by regulating microtubule dynamics (Hur and Zhou, 2010). Besides Akt, PIP3 also provide a signal for membrane recruitment and activation of a number of small GTPase regulators, particularly GEFs (Guanine nucleotide Exchange Factors) and GAPs (GTPase Activating Proteins) of the Rho and Arf families of GTPases (Krugmann et al., 2002; Welch et al., 2002). In this manner PI3K, can regulate the activation state of Rho and Arf family GTPases, thereby influencing cytoskeletal and vesicular trafficking events at the membrane crucial for almost all aspects of neuronal polarity including migration, neurite outgrowth as well as axon and dendrite specifications. PTEN inhibits the signaling output of PI3Ks by dephosphorylating PIP3 back to PIP2 (PI(4,5)P2). The numerous modes of activation of PI3K by upstream signals and growth factors are not depicted. **(B)** PTEN is formed of 403 amino acids and its structure displays an N-terminal PIP2 binding motif (PBM), a phosphatase domain, a calcium-independent lipid-binding C2 domain, and a flexible C-terminal tail (Cter-tail) followed by a PDZ-binding motif (PDZ) (Lee et al., 1999; Shi et al., 2012; Song et al., 2012). The phosphatase and C2 domains form a minimal structural unit that is capable of dephosphorylating PIP3 (Lee et al., 1999). Interestingly, highly unstructured elements such as the PBM, parts of the C2 domain and the C-terminal tail have important implications for the regulation of PTEN (Lee et al., 1999; Malaney et al., 2013). The phosphatase domain bears the P-loop signature motif of dual-specificity tyrosine phosphatases (^123^HCXXGXXR^130^) with Cys124 being the active site residue. Mutation of Cys124 to Ser renders PTEN inactive and this form of PTEN has been important to determine whether PTEN functions depend on its phosphatase activity (Myers et al., 1998). Interestingly, PTEN can also function as a protein phosphatase and putative protein substrates have been proposed in seminal studies (e.g., Tamura et al., 1998). The PTEN protein phosphatase activity can be equally important for autoregulation of PTEN and some PTEN functions pertinent to its tumor suppressive role, at least under certain settings (Tibarewal et al., 2012; Bassi et al., 2013). The positions of PTEN posttranslational modifications (ubiquitination and phosphorylation) as well as catalytic and membrane binding features (P-loop and CBR3-loop) that are discussed in the text are indicated.

PTEN functions predominately by directly antagonizing the activity of PI3K class I heterodimeric enzymes at the plasma membrane (Figure 1A). Class I PI3Ks are activated by a broad array of growth factors, components of the extracellular matrix, and G-protein coupled receptor (GPCR) agonists (Hawkins et al., 2006; Vanhaesebroeck et al., 2010). PI3Ks synthesize phosphatidylinositol 3,4,5-trisphosphate (PIP3) from phosphatidylinositol 4,5-bisphosphate (PIP2), a phosphoinositide that is particularly enriched in the inner leaflet of the plasma membrane of all mammalian cells. In fact, it can be argued that this paradigm of extracellular signal/agonist-induced activation of PI3Ks represents perhaps the most prevalent signal transduction event associated with mammalian cell-surface receptor activation (Hawkins et al., 2006). Details concerning historical perspectives, mechanisms of activation, feedback regulatory mechanisms and crosstalk with other signaling modules, isoform-specific roles and downstream effectors of this prominent signaling pathway can be found in recent excellent reviews (Hawkins et al., 2006; Vanhaesebroeck et al., 2010, 2012). For the purpose of this review we will only highlight certain aspects of PI3K signaling that are key to understand the implications of PTEN function, particularly in neuronal cells.

### Basic principles of PI3K signaling

The major and primary output of PI3K signaling is PIP3. Its rapid synthesis upon PI3K activation coordinates the localization and function of multiple effector proteins, which utilize specific lipid-binding domains, such as PH (pleckstrin homology) domains, to recognize and bind this lipid on the plasma membrane. As suited for a second messenger, PIP3 is also subject to tight regulation by specific phosphoinositide phosphatases. PTEN is one of the most important phosphatases because it directly antagonizes the PI3K reaction by its 3-phosphatase activity and dephosphorylates PIP3 back to PIP2. In this sense, PTEN represents a brake built into PI3K signaling and its absence or dysfunction results in the constitutive and unregulated elevation of the signaling output of PI3Ks. Alternatively, PIP3 can be dephosphorylated by type II phosphoinositide 5-phosphatases like SH2-domain containing inositol 5-phosphatases 1 and 2 (SHIP1 and SHIP2) (Leslie et al., 2008). These enzymes generate PI3,4P2 (phosphatidylinositol 3,4-bisphosphate), which can be viewed as a secondary signaling output of PI3K. PI3,4P2 binds to sets of effector proteins that overlap with PIP3 effectors but also recruits others specifically (Vanhaesebroeck et al., 2010). Prominent PIP3 effectors and the pathways that relay PI3K signaling, as well as the domain structure of PTEN are briefly presented in the legend of Figure 1.

### Basic principles of PTEN regulation

PTEN expression and activity is tightly regulated at almost all possible levels: transcriptionally, translationally, and posttranslationally (Shi et al., 2012; Song et al., 2012). Perhaps the most relevant mode of PTEN regulation, however, is through posttranslational modifications and interaction with other proteins. PTEN is modified by phosphorylation, acetylation, oxidation, S-nitrosylation, ubiquitination, and sumoylation (Shi et al., 2012; Song et al., 2012; Bassi et al., 2013). A significant body of evidence now suggests that these posttranslational modifications may influence the phosphatase activity, the binding to the membrane, the localization to subcellular compartments, or the interaction with binding partners. For PTEN to fulfill its broad mechanisms of action ranging from regulation of proliferation to the establishment of defined neuronal circuits, it needs to be finely tuned both temporally and spatially. Interaction with PIP2/PIP3-rich plasma membrane domains is apparently an important feature of PTEN as a PIP3 phosphatase and we begin to form a comprehensive model of how this interaction is regulated. In addition, a surprising aspect of many recent findings has been that PTEN can be found in different cellular organelles. In this review, we focus specifically on the mechanisms of PTEN's association and recruitment to membranes, the function of PTEN in different subcellular compartments, how PTEN localizes in these specialized regions and why understanding the regulation of PTEN in these defined localized areas has become increasingly important in the context of neurological diseases.

## Mechanisms of PTEN cell membrane association and recruitment

### Phospholipid-specific functions during association of PTEN with cell membranes

PTEN has been characterized as a highly plastic protein with strong intramolecular interactions and conformational changes that result from posttranslational modifications but also can occur as a consequence of interactions with other proteins or lipids. Under basal conditions, small concentrations of PTEN dynamically interact with the plasma membrane, a process that is abrogated following deletion of the N-terminal PBM (Vazquez et al., 2006). Distinct roles for the acidic phospholipids phosphatidylserine (PS), PIP2, and PIP3 in regulating PTEN at the membrane have been demonstrated. Whilst PIP2 interaction with the PBM seems to be the primary determinant of PTEN's plasma membrane localization (Rahdar et al., 2009; Shenoy et al., 2012), PS has been shown to support electrostatic attraction within the protein-membrane interface primarily via the C2 domain (Shenoy et al., 2012). Thus, PS and PIP2 appear to act synergistically in driving PTEN membrane interaction (Shenoy et al., 2012). PIP3, on the other hand, which binds to the phosphatase domain, does not appear to be a key determinant of PTEN's membrane association, at least when presented at physiological concentrations (Shenoy et al., 2012).

### Regulation of PTEN membrane association by conformational changes

To date, the model that best explains the accessibility of PTEN to membranes suggests the presence of PTEN in two basic conformations, one of which is closed and the other open (Vazquez and Devreotes, 2006). The closed conformation is induced by an intramolecular association of the PTEN C-tail and part of the PTEN C2 domain (Figure 1B). Induction of the closed conformation is a process that occurs preferentially following phosphorylation of the PTEN C-tail (Odriozola et al., 2007; Rahdar et al., 2009; Ross and Gericke, 2009; Shenoy et al., 2012; Bolduc et al., 2013). Consequently, the open conformation of PTEN is thought to be unphosphorylated on the C-tail cluster, or at least to be less so. PTEN interactions with membranous PS and PIP2 may also induce conformational changes (Redfern et al., 2008; Shenoy et al., 2012), and it has been proposed that other secondary posttranslational modifications, such as sumoylation may provide a positive signal for PTEN membrane association (Huang et al., 2012). Therefore, levels of phosphorylation of the PTEN C-tail cluster at S/T 380–385, although highly informative, may not always faithfully report on PTEN's activation state.

### Regulation of PTEN membrane recruitment by interacting proteins

The recruitment of PTEN to membranes is further dictated through association with other proteins (Song et al., 2012). In this respect, transmembrane, peripheral membrane, and membrane-associated scaffold proteins that interact with PTEN are particularly important in positioning PTEN within close proximity to the membrane. In effect, such protein-protein interactions prime PTEN function toward membranous substrates (Sumitomo et al., 2004; Wu et al., 2007). Examples include the transmembrane protein NEP (Neutral Endopeptidase), which binds the phosphorylated PTEN C-tail via a highly positively charged cytosolic domain and recruits the phosphatase to the plasma membrane, enhancing both PTEN protein stability and activity (Sumitomo et al., 2004). PTEN has been also shown to interact with plasma membrane receptors of the tyrosine kinase and G protein-coupled receptor families, although it is not always clear if these interactions are direct (Sanchez et al., 2005; Fenton et al., 2012; Cao et al., 2013). In addition, several PDZ domain-containing proteins interact with the C-terminal PTEN PDZ-binding motif and recruit PTEN to the plasma membrane (Wu et al., 2000; Von Stein et al., 2005; Jurado et al., 2010; Molina et al., 2012; Terrien et al., 2012). PTEN-PDZ domain interactions may also serve in shifting PTEN into the open “active” conformation by sequestering the PTEN C-tail away from interacting with the C2-domain and unmasking the PTEN membrane-binding regions (Vazquez and Devreotes, 2006). However, PTEN recruitment to membrane proteins may also provide efficient “off”-switches for PIP3 hydrolysis by sequestering PTEN away from its substrate.

Membrane associated PTEN interacting proteins have further been described as PTEN adaptor proteins, since several of these facilitate PTEN's recruitment to activated receptors and places of highly restricted production of PIP3. Examples include NHERFs (Na(+)/H(+) exchanger regulatory factors) or β -arrestins, which recruit PTEN to growth factor receptors (Lima-Fernandes et al., 2011; Molina et al., 2012). Interestingly, NHERF1, besides binding PTEN, also associates with the prominent Akt phosphatase PHLPP1 (PH domain and Leucine-rich repeat Protein Phosphatase), which leads to a synergistic attenuation of both PI3K-mediated PIP3 production as well as downstream Akt pathway activity (Molina et al., 2012). Efficient PI3K signaling feedback regulation during acute receptor tyrosine kinase signaling can be achieved by co-recruitment of PTEN with the regulatory subunit of PI3K class I enzymes, p85α (Chagpar et al., 2010). In some of the interactions, the recruitment of PTEN to signaling complexes appears to be agonist-induced (Sanchez et al., 2005; Fenton et al., 2012). Therefore, a clear distinction can be made between PTEN-membrane protein interactions that appear to be constitutive and interactions that are agonist-induced. One can only surmise the latter to be spatially restricted to specific sites of active PI3K signaling.

### PTEN, lipid rafts, and associated microdomains

A further mode of membrane PTEN recruitment is orchestrated by lipid rafts or detergent-resistant membrane fractions in different cells such as cortical neurons, oligodendroglioma, and pheochromocytoma cell lines (Cheung et al., 2004; Goswami et al., 2005; Choy et al., 2006). Lipid rafts are specialized membrane microdomains involved in the compartmentalization of cellular processes and act as organizing centers for the assembly of signaling molecules. Two types of lipid rafts have been proposed: planar lipid rafts and caveolae. Planar rafts are defined as being continuous with the plane of the plasma membrane, whilst caveolae invaginate with the help of caveolin proteins. Planar rafts contain flotillin and are found in neurons where caveolae are absent. Both types of lipid rafts demonstrate similar lipid compositions that are enriched in cholesterol and sphingolipids. Lipid raft association of PTEN in neurons appears to be related to apoptosis either induced by lactacystin (Choy et al., 2006), or by treatment with N-acetylsphingosine (C2-ceramide) (Goswami et al., 2005). On the one hand, PTEN's recruitment to flotillin-rich plasma membrane domains was demonstrated during lactacystin-induced apoptosis in cortical neurons and verified by immunogold-TEM (Choy et al., 2006). On the other hand, PTEN has been found to associate with caveolin in immunoprecipitation experiments, which could account for recruitment to caveolin-rich subcellular fractions in caveolae-positive cell types (Caselli et al., 2002). In another study, PTEN was found to localize into the non-raft region of the plasma membrane (Gao et al., 2011). This localization was essential for allowing proper and robust activation of PDK1 and Akt in response to occurrence of growth factors receptor activation in the lipid raft region (Gao et al., 2011). Indeed, forced localization of constitutively activated PTEN to lipid rafts, or treatment with C2-ceramide (Goswami et al., 2005), was found to prevent growth factor-induced lipid raft restricted activation of PDK1 and Akt (Gao et al., 2011). Together, these studies suggest that activators and inhibitors of the PI3K pathway might be segregated to distinct sub-compartments at the level of the plasma membrane in dependence of the lipid raft composition. Nevertheless, the exact targeting mechanisms that segregate neuronal PTEN from lipid rafts, or that recruit PTEN into lipid rafts upon signaling, are currently unknown.

### PTEN membrane association in neurons

**Figure 2:**
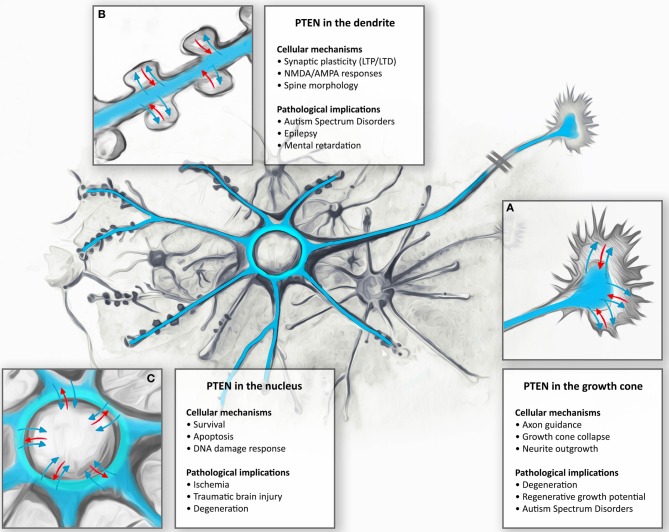
**Subcellular targeting and dynamic regulation of PTEN in neurons: Schematic illustration demonstrating dynamic distributions of PTEN in different neuronal compartments. (A)** During neuronal development, PTEN is enriched in the axons and dendrites. Here, PTEN is thought to function in the regulation of growth cone dynamics during axonal navigation, in particular by inhibiting neurite outgrowth or mediating growth cone collapse responses. Consequently, PTEN-loss results in increased regenerative growth of axons in spinal cord injury models as well as protecting neurons during degeneration. **(B)** In mature CNS neurons PTEN is found in the dendrite. During NMDAR-dependent dendritic spine plasticity (long-term depression, LTD), PTEN translocates deep into the spine and anchors to the postsynaptic density. PTEN, by targeting membranous PIP3, also contributes to the dynamic changes in spine morphology during synapse development and plasticity. These synapse specific functions are thought to contribute to neurodevelopmental disorders such as autism, epilepsy, and mental retardation. **(C)** Nuclear PTEN has been reported to mediate neuronal survival or specifically induces apoptotic responses. Movement to the nucleus has been reported to occur during ischemia, traumatic brain injury, and degeneration; however, specific functions of these translocations are not clear.

**Figure 3:**
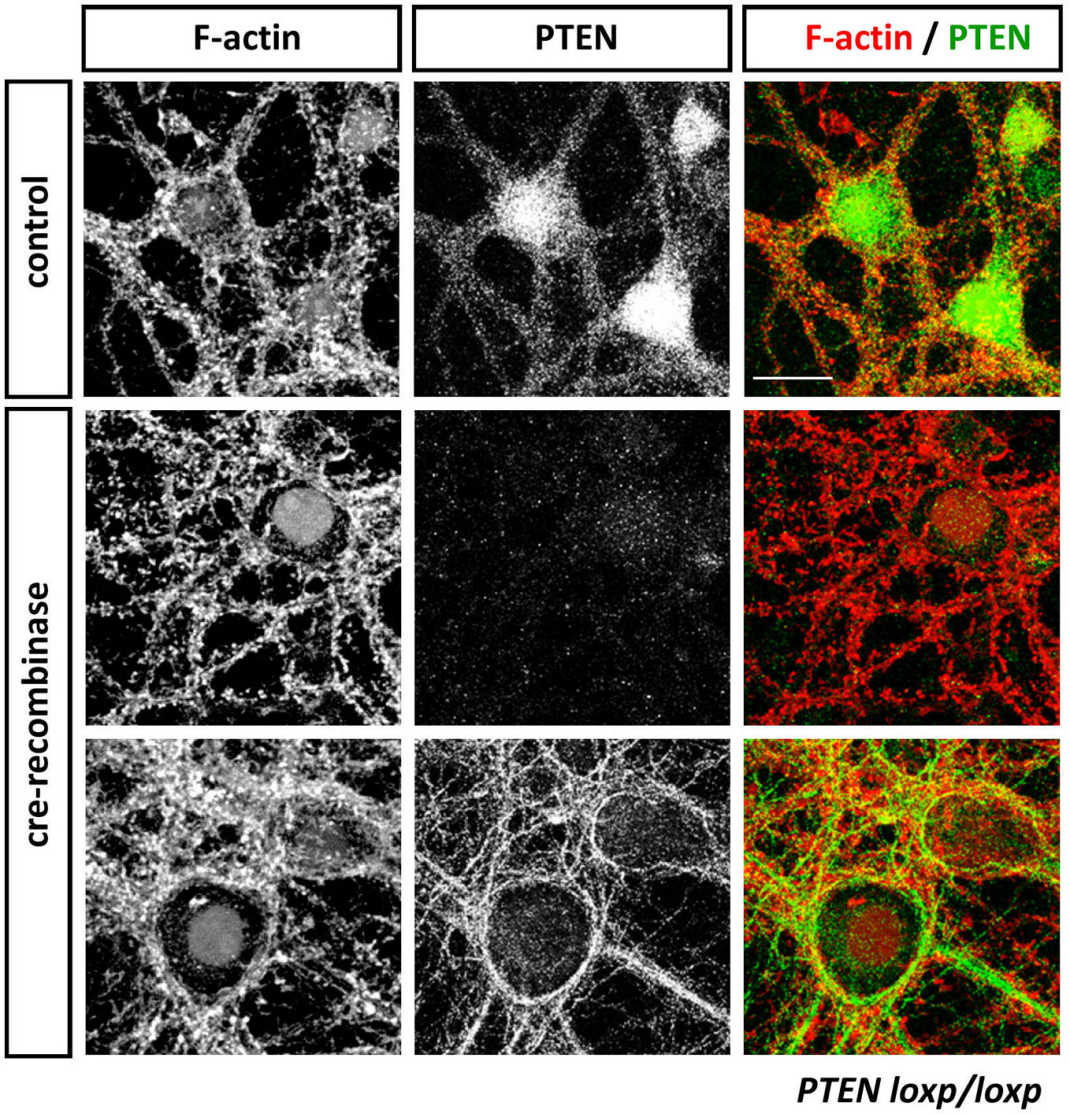
**Anti-PTEN antibodies do not always faithfully report on subcellular distributions of PTEN**. Cortical neurons isolated from *PTEN*^*flox*/*flox*^ mice were transduced with control or Cre-expressing viruses at 13 days *in vitro* (DIV). Neurons were fixed with 4% parafolmaldehyde at 25 DIV, permeabilized with 0.1% Triton X-100 and stained with Phalloidin to visualize F-actin and anti-PTEN antibodies 138G6 (rabbit monoclonal from Cell Signaling Technology) and A2B1 (mouse monoclonal from Santa Cruz Biotechnogy). PTEN as detected with 138G6, (1:400 dilution) is highly enriched in the neuronal soma and dendrites, but it is largely absent in dendritic spines (first row). Cre-mediated recombination induced a PTEN-loss that resulted in well-established morphological changes in neuronal morphology including, for example, hypertrophy of the soma. In Cre-treated neurons, PTEN labeling using 138G6 was absent (second row), whilst the mouse A2B1 anti-PTEN antibody (third row) still retained strong labeling (although used at a high dilution of 1:1000). Note that the faint nuclear staining in control and Cre-recombinase treated neurons in F-actin images is due to nuclear RFP and RFP-Cre expressed, respectively, in these cells. Scale bar = 20 μm.

PTEN and its associated PI3K signaling pathway have been shown to be particularly relevant in neurons during the control of cell growth, division, survival, and differentiation, in order to produce highly polarized neuronal morphologies with exquisite specializations such as growth cones and synapses. The proper localization of PTEN at the membrane is a key factor in the establishment of a PIP3/PIP2 gradient and the recruitment of important components necessary for the formation of growth cones and dendritic spines. PTEN is present in most, if not all, neurites during early neurite outgrowth, and in axons and dendrites in mature neurons. In particular, PTEN seems to be maintained at the microtubule-rich, central domain of growth cones with relatively low levels seen in the peripheral growth cone domain with its actin-rich filopodia and lamellipodia (Chadborn et al., 2006; Kreis et al., 2013; see Figure 2). Similarly, whilst PTEN-loss induces dramatic rearrangements in dendritic spine morphology and affects synaptic transmission, endogenous levels of PTEN are barely detectable in this neuronal compartment (Figure 3; Fraser et al., 2008; Kreis et al., 2010, 2013). These observations suggest that, specifically in neurons, PTEN may be sequestered away from the cell membrane and selectively recruited to the membrane under certain conditions. PTEN localization at the growth cone membrane has been shown to be mainly involved in the regulation of a PIP3 pool to induce chemorepulsion. On one hand, during Sema3A-induced growth cone collapse, neurite retraction, and chemorepulsion in dorsal root ganglion (DRG) growth cones, PTEN is recruited transiently and accumulates at the membrane, leading to decreases in PIP3 levels and an antagonistic effect on the PI3K signaling pathway (Chadborn et al., 2006). Along this vein, it has been suggested that PTEN is required for the activity of a number of established neurite outgrowth inhibitors, such as Sema3A, Sema4D. and Myelin-associated glycoprotein (MAG) in DRGs, hippocampal, cortical, and spinal neurons (Chadborn et al., 2006; Oinuma et al., 2010; Perdigoto et al., 2011; Henle et al., 2013). On the other hand, polarized increases in PIP3 at the leading edge of growth cones is an essential element of axonal growth and growth cone attraction (Henle et al., 2011). Accumulation of PIP3 has been seen also in axonal microdomains proximal to the growth cone. These PIP3 microdomains are induced by NGF (Nerve growth factor) in DRG neurons and they seem to be necessary for the formation of axonal F-actin patches that support the generation of axonal filopodia (Ketschek and Gallo, 2010). Similarly, optogenetic control of PIP3 accumulation can induce the formation of active mobile F-actin lamellipodia-like structures in growth cones or along proximal axonal segments (Kakumoto and Nakata, 2013). These PIP3-rich membrane domains can be endocytosed, restricting downstream signaling components and thus leading to the modification of the size and function of the growth cone. Local accumulation of PIP3 is also important for axonal growth. Under basal conditions on a permissive laminin substrate, PIP3 levels are concentrated within the peripheral domain and fluctuate stochastically (Henle et al., 2013). However, upon exposure to chemoattractive growth guidance cues, PIP3 accumulation occurs in a polarized fashion toward the source of the cue (Henle et al., 2011). Thus, a feasible mechanism of PTEN functioning in neurons maybe to reinforce or re-sensitize PI3K-dependent signaling during growth factor promoted axonal elongation, as well as mediating axonal growth inhibition (Figure 2).

Using PIP3-specific Fluorescence resonance energy transfer (FRET) sensors to study the role of PIP3 at the synapse compartment, Ueda and Hayashi showed that PIP3 is primarily enriched in spines and not in dendritic shafts (Ueda and Hayashi, 2013). Upon chemical inhibition of PTEN, PIP3 was seen to accumulate preferentially in dendritic shafts rather than in spines, indicating that PTEN activity in the shaft region is higher while the opposite appears to apply for PI3K activity (Ueda and Hayashi, 2013). Small amounts of PTEN are present in the cytosolic compartment of spine heads, and in the plasma membrane away from the postsynaptic density (PSD) (Jurado et al., 2010). However, following NMDAR (N-methyl-D-aspartate receptor) stimulation, PTEN can be further enriched in the dendritic spine and anchored to the PSD primarily via PDZ-dependent interactions with PSD-95 (Jurado et al., 2010). Analysis of GFP-PTEN mobility in the spine has suggested that at least half of the PTEN spine pool is stable under basal conditions but, upon acute NMDAR activation there is increased mobility and eventually retention of PTEN into the spine (Jurado et al., 2010). Interestingly, deletion of the PDZ-binding domain of PTEN still results in increased mobility but abolishes the retention of PTEN into the spine (Jurado et al., 2010). It is conceivable that there is a basal diffusion of a small PTEN pool into spine that can then be retained and further targeted to the PSD in an activity-dependent manner. Thus, it appears that regulated PTEN localization in the spine and the PSD may be important for synapse-specific modulation of PIP3 signaling during synaptic plasticity. The recruitment and activation of PTEN at the membrane of neurons are important elements to the establishment of a localized PIP3/PIP2 balance necessary for specialized regions of the neurons to form and function in a required way. Other PIP3-independent roles of PTEN may also be important in this neuronal compartment, together they converge to allow the correct functioning of the synapse.

## Evidence for PTEN association with internal membrane compartments

Based on the characteristic enrichments of PIP2 and PS in the inner leaflet of plasma membranes, the activity of membrane-bound PTEN is mostly confined to this compartment. However, a unique feature of PTEN is its ability to interact also with internal membrane containing organelles. The specific roles of PTEN at these membrane systems remains poorly understood. Because of the established link of these organelles to the pathology of different neurological conditions, we briefly review these relatively recent findings here.

### PTEN and mitochondria membranes

PTEN localizes to mitochondria (Zhu et al., 2006; Zu et al., 2011; Bononi et al., 2013). In primary rat hippocampal neurons, PTEN's association to mitochondria membranes has been demonstrated by both immunohistochemistry and biochemical fractionation experiments, a localization that has been shown to increase during apoptotic stimuli, for example using staurosporine (Zhu et al., 2006). PTEN was found to interact with a member of the Bcl-2 (B-cell lymphoma 2) gene family, BAX, in co-immunoprecipitation experiments and this protein-protein interaction has been attributed to regulating the recruitment of PTEN to the outer mitochondrial membrane, at least during the initial phases of the response. A similar association of PTEN with Bax and mitochondria has been described recently in myocytes during ischemia-reperfusion (Zu et al., 2011). Following induction with apoptotic stimuli, BAX becomes organelle membrane-associated, in particular, with mitochondrial membranes. On this basis, PTEN's transient association with BAX has been connected to mitochondria-dependent apoptotic death. But, as yet, specific mechanistic insight into a proposed PTEN connection has not been achieved.

### PTEN and endoplasmic reticulum membranes

A recent study demonstrated the presence of PTEN at the endoplasmic reticulum (ER) with apparent specific enrichment in mitochondria-associated membranes (MAMs) (Bononi et al., 2013). MAMs are distinct ER domains that support physical interaction between the ER and mitochondria and that are thought to be essential for efficient supply of Ca^2+^ from the ER to mitochondria (Fujimoto and Hayashi, 2011). Accordingly, they play major roles in both Ca^2+^-regulated mitochondrial bioenergetics and sensitivity to apoptosis, amongst other processes (Fujimoto and Hayashi, 2011). Sub-cellular localizations of PTEN to the ER and to MAMs were verified by immunostaining and biochemical fractionation experiments. An interaction of PTEN, as well as of Akt, with the ER inositol 1,4,5-trisphosphate receptor (InsP3R) at the ER-MAM membranes—the membrane glycoprotein complex that acts as the Ca^2+^-channel activated by inositol trisphosphate—was detected (Bononi et al., 2013). Importantly, apoptotic stimuli enhanced the recruitment of PTEN to these InsP3R complexes, leading to reduced Akt activity and Akt-dependent InsP3R phosphorylation and resulting in an increased Ca^2+^ release (Bononi et al., 2013). Specific targeting of PTEN to the ER was found to be sufficient to enhance ER-to-mitochondria Ca^2+^ transfer and sensitivity to apoptosis. Notably, this mechanism was dependent on the PTEN protein phosphatase activity and not the PTEN lipid phosphatase activity (Bononi et al., 2013). Although the mechanism of PTEN's recruitment to MAMs and non-MAM ER membranes remains unclear, it is likely that the PTEN-InsP3Rs association is involved. Whilst small PIP3 pools are present in ER membranes and mitochondria (Lindsay et al., 2006), no evidence has been provided that suggests PTEN actively regulates these pools (Bononi et al., 2013). Given the proximity of MAMs to mitochondria, it is also possible that some of the mitochondria-associated PTEN functions could be related to its localization in the MAMs. Interestingly, MAMs have been detected in neurons and synaptosomes and are important for neuronal ER-mitochondria communication as well as neuronal survival (Hedskog et al., 2013). Thus, PTEN targeted to ER-MAMs in neurons could regulate mitochondria functions such as Ca^2+^ homeostasis or induce apoptotic signaling as implicated, for example, in neurodegenerative diseases such as Alzheimer's disease (AD).

### PTEN, exosomes, and PTEN-long isoform secretion

Exosomes are bona fide secreted membrane organelles that contain cytosolic or endosomal proteins, and that are thought to provide essential intercellular communication. In this manner, exosomes serve as mediators to convey proteins, lipids and genetic information from one cell to another. Exosomes are formed in the cell in multivesicular endosomes that can fuse to the membrane to release their contents (Raposo and Stoorvogel, 2013). Recently, it has been shown that PTEN can be secreted in exosomes, exported in the extracellular compartment and taken up by recipient cells. This sequence of events has raised the exciting possibility that PTEN has non-cell-autonomous functions (Putz et al., 2012). Exosome-mediated PTEN secretion has been shown to require Ndfip (Nedd4 family interacting protein-1), an adaptor protein for E3 ubiquitin ligases, and an intact ubiquitination residue at PTEN K13 (Putz et al., 2012). PTEN loaded exosomes can inhibit the PI3K signaling pathway in recipient cells and decrease cellular proliferation (Putz et al., 2012). A further cellular strategy shown to generate secreted forms of PTEN is the presence of an additional sequence of 173 amino acids at the PTEN N-terminus (Hopkins et al., 2013). This PTEN-long phosphatase, translated from an alternative initiation codon upstream of the normal initiation codon, contains a secretion signal and a cell penetrating sequence and has the property to interact with cell surface proteins. When added as exogenous agent to cells in culture or injected intraperitoneally in mice, PTEN-long was able to decrease the activity of the PI3K signaling pathway. In addition, when injected in tumor mouse models, PTEN-long was able to induce tumor regression (Hopkins et al., 2013).

The mechanisms underlying PTEN secretion require further exploration and may turn out to depend on the cell status or the cell type. Exosomes have been detected in neurons and glial cells, suggesting that they may serve as regulators of synaptic plasticity or participate in the propagation of pathological proteins responsible for neurodegenerative diseases (Chivet et al., 2013; Frühbeis et al., 2013). PTEN-loaded exosomes or PTEN-long expression could thus allow different intercellular exchanges within different brain regions, thereby modulating, for example, local changes in PI3K-mediated synaptic plasticity. Both forms of secreted PTEN appear to be physiologically and clinically significant as they can be readily detected in human blood (Gabriel et al., 2013; Hopkins et al., 2013).

## Nuclear functions of PTEN

Despite its well-established role in antagonizing PI3K signaling at the plasma membrane, PTEN has also been characterized as present in the nucleus of a number of cells, including fully differentiated neurons (Gimm et al., 2000; Lachyankar et al., 2000; Chadborn et al., 2006) (Figure 3). It appears that nuclear localization of PTEN is a dynamic process that correlates with cell cycle progression and/or the cellular differentiation state, and that it may be highly context dependent (Lian and Di Cristofano, 2005; Planchon et al., 2008; Song et al., 2012). In several cancer types, nuclear localization is a contributory factor to PTEN's tumor suppressor activity, for example, by maintaining chromosomal integrity (Shen et al., 2007), and regulating cellular survival (Chung et al., 2005; Gil et al., 2006), cell cycle, and DNA repair responses (Shen et al., 2007; Song et al., 2011; Bassi et al., 2013). Nuclear PTEN can also directly interact with p53 and alter transcriptional activity (Freeman et al., 2003). Importantly, recent studies have highlighted the importance of nuclear PTEN also in neurons, particularly in the context of neuronal survival (Figure 2). PTEN has been found to translocate to the nucleus during ischemia, traumatic brain injury (TBI) and during NMDA-induced excitotoxicity (Howitt et al., 2012; Goh et al., 2013; Zhang et al., 2013). In NMDA-challenged cortical neurons, translocation of PTEN to the nucleus is a delayed event, peaking at 6–9 h after treatment and with a decline after 24 h (Zhang et al., 2013). However, a persisted localization of PTEN in the nucleus of cells after 24 h has been observed in TBI models in cortical neurons (Goh et al., 2013). Whether nuclear PTEN functions as a pro-survival protein or whether it contributes to cell death remains controversial. For example, nuclear translocation of PTEN following TBI or cerebral ischemia (Howitt et al., 2012; Goh et al., 2013) does not seem to correlate with increased apoptosis *in vivo*; rather, it has been proposed that it might be a generic neuroprotective response of stressed neurons (Goh et al., 2013). Yet, in NMDA-challenged cortical neurons, inhibition of PTEN's nuclear translocation by a peptide designed to antagonize K13-ubiquitination of PTEN appears to be neuroprotective (Zhang et al., 2013). Further experiments are urgently needed to clarify the functions of nuclear PTEN in neurons since genetic or pharmacological PTEN inhibition is discussed as a promising approach to increasing neuroprotection in various settings (Ding et al., 2013; Mao et al., 2013). But how does PTEN function in the nucleus? Currently, there seems to be substantial evidence that at least some (if not most) of nuclear functions do not directly involve PTEN's PIP3 phosphatase activity (Song et al., 2012). PIP3 is present in the nucleus, as well as different PI3K isoforms and activated Akt, yet a detailed ultrastructural study suggested that the nuclear pool of PIP3 is not sensitive to hydrolysis by PTEN (Lindsay et al., 2006). Furthermore, given that PTEN's activity toward PIP3 requires structural interactions with membranes, it would be hard to reconcile this taking place in membrane-free, subnuclear compartments, where most of the nuclear PIP3 pool is located. Interestingly, a recent study suggested the possibility of different conformations adapted by cytosolic and nuclear PTEN, which may suggest phosphatase-independent functions for nuclear PTEN (Moncalero et al., 2011). Indeed, several nuclear PTEN functions that are relevant to its tumor suppressing role, such as control of the activity of the Anaphase Promoting Complex/Cyclosome (APC/C) and maintaining chromosomal integrity, do not require its lipid nor its protein phosphatase activity (Shen et al., 2007; Song et al., 2011). Other nuclear PTEN functions, however, have been shown to require at least its protein phosphatase activity (Bassi et al., 2013). Interestingly, NMDA treatment of cortical neurons, leads to nuclear PTEN translocation, resulting in decreases in PIP3 levels and Akt phosphorylation in this compartment (Zhang et al., 2013). However, whether nuclear PTEN in neurons is indeed active against nuclear PIP3 and thus regulates survival by inhibiting Akt remains to be tested.

### Nuclear targeting of PTEN

PTEN interacting proteins like MVP (Major Vault protein), the GTPase Ran, or the protein PNUFTS (a nuclear targeting subunit of PP1) may have a function in transporting or sequestering PTEN to the nucleus (Chung et al., 2005; Gil et al., 2006; Kavela et al., 2013). Nevertheless, the mechanisms of PTEN nucleo-cytoplasmic shuttling appear to primarily involve posttranslational modifications such as monoubiquitination or sumorylation (Trotman et al., 2007; Bassi et al., 2013). The phosphorylation of the C-terminus of PTEN has also been suggested to influence the cytoplasmic and nuclear localization of PTEN, which may be related to the conformation of PTEN modifying its potential to be ubiquitinated or to localize to membranes enriched in ubiquitin ligases (Vazquez et al., 2001; Gil et al., 2006; Maccario et al., 2010). Two PTEN monoubiquitination sites, K289 and K13, have been found to contribute to the localization of PTEN to the nucleus (Trotman et al., 2007; González-Santamaría et al., 2012; Zhang et al., 2013). Particularly in neurons, K13 alone appears to be important for PTEN nuclear localization (Zhang et al., 2013). However, K13 is also important for membrane association of PTEN as it is part of the PBM region (Walker et al., 2004; Figure 1B). Early studies indicated that the E3 ubiquitin ligase Nedd4 (neural precursor cell-expressed developmentally downregulated gene 4) can regulate monoubiquitination of PTEN and regulate PTEN's import into the nucleus (Trotman et al., 2007). Ndfip1, an adaptor for Nedd4-mediated ubiquitination and a direct binding partner of PTEN (Mund and Pelham, 2010) is also necessary for nuclear localization of PTEN in neurons (Howitt et al., 2012). A PTEN-deubiquitinating enzyme, HAUSP (herpesvirus-associated ubiquitin-specific protease) may serve in the deubiquitination of PTEN and its shuttling out of the nucleus (Song et al., 2008). To date, most Nedd4/PTEN studies in neurons have focused on the role of polyubiquitination in controlling PTEN abundance by the ubiquitin-proteasome system and identified that PTEN protein levels can be correlated with changes in survival or axonal growth and branching (Christie et al., 2010; Drinjakovic et al., 2010; Kwak et al., 2010).

## PTEN localization and the cytoskeleton

In developing neurons, PI3K, PTEN, and Akt downstream signaling pathways are essential for most aspects of neuronal polarity, such as neurite outgrowth, axon and dendrite specification, growth cone guidance and migration (Waite and Eickholt, 2010). The intimate relationship of phosphoinositides and their impact on actin cytoskeleton dynamics stems from the fact that many proteins that are known to affect the actin-based cytoskeleton are acutely regulated by PIP2 and/or PIP3 (Yin and Janmey, 2003; Di Paolo and De Camilli, 2006). Indeed, PIP3 is regarded as a master regulator of Rho and Arf family-specific GEFs/GAPs (Hawkins et al., 2006) and its turnover is linked to initiation of cell migration and actin cytoskeleton reorganization, including, for example, the formation of structures like F-actin patches, lamellipodia and filopodia (Gallo, 2013; Kakumoto and Nakata, 2013; Karunarathne et al., 2013). The local balance between PIP2 and PIP3 may be essential during initiation and emergence of filopodia, as suggested by axonal filopodia biogenesis models (Gallo, 2013). Initial localized synthesis of PIP3 in membrane microdomains precedes and marks the position of actin patches (Ketschek and Gallo, 2010; Spillane et al., 2011). As PIP3 levels decline, increases in PIP2 may recruit the molecular machinery driving the emergence of a filopodium from the actin patch (Gallo, 2013). According to this model, dynamic hydrolysis of PIP3 to PIP2 by PTEN might be instrumental. However, analysis by immunostaining or live imaging of fluorescent PTEN constructs has rarely shown constitutive localization of PTEN in prominent F-actin structures, for example in the lamellipodia-rich leading edge of chemoattracting cells or in the peripheral zone of migrating growth cones (Li et al., 2005; Chadborn et al., 2006; Kreis et al., 2010). This is usually regarded as a sequestration of PTEN away from these sites in order to allow a steep PIP3 gradient to be formed and maintained, particularly during cell migration. In the case of growth cones and processes of neurons and neuronal cells, PTEN instead localizes rather prominently with microtubules (Chadborn et al., 2006) and recruitment to specific F-actin rich sub-plasmalemma domains may be driven by active transport mechanisms (Van Diepen et al., 2009; Kreis et al., 2010).

PTEN function has also been shown to be controlled by regulators of F-actin reorganization such as Rho family GTPases, Rac and Rho. P-Rex2a, a prominent Rac activator that is activated by PIP3, inhibits PTEN PIP3 phosphatase activity by direct interaction (Fine et al., 2009; Hodakoski et al., 2014), while active RhoA is thought to stimulate PTEN's PIP3 phosphatase activity, by ROCK-mediated phosphorylation of PTEN (Li et al., 2005). There is further a substantial degree of crosstalks and feedback loops embedded within the PI3K-PTEN-Rac/Rho GTPase network. Membranous PIP3, is able to activate RacGEFs, yet Rac is unique amongst Rho family GTPases in that it can directly activate specifically p110β PI3K, the only Ras-insensitive PI3K class I isoform (Fritsch et al., 2013). Furthermore, p110δ PI3K can inhibit PTEN via RhoA/ROCK (Rho-associated protein kinase) (Eickholt et al., 2007; Papakonstanti et al., 2007). Another feature that adds complexity to the relationship of PTEN and the F-actin cytoskeleton is due to the fact that PIP3 can also be metabolized to PI3,4P2 by SHIP2 and other 5-phosphatases (Leslie et al., 2008). PI(3,4)P2 by itself is involved in many aspects of F-actin cytoskeleton reorganization by interaction with its specific effectors such as lamellipodin (Krause et al., 2004; Yoshinaga et al., 2012). Thus, PTEN may work in concert with 5-phosphatases in order to achieve precise regulation of the timing and extent of PI3K-generated PIP3 and PI3,4P2 at active sites of F-actin remodeling during neurite extension or growth cone dynamics (Aoki et al., 2007).

PTEN by itself interacts with actin cytoskeleton structural, accessory, or regulatory proteins, although, in most cases, the direct effects of PTEN activity or actin cytoskeleton function is not known. For example, PTEN was recently identified as part of a plasma membrane-associated actin remodeling complex composed of actin, gelsolin, and EPLIN (Epithelial Protein Lost In Neoplasm) (Kim et al., 2011b). Although it's unclear whether PTEN directly regulates the activity of the proteins in this complex, this association is proposed to be functional in regulating cell size checkpoint control in irradiated cells (Kim et al., 2011b). Early studies have shown that PTEN can regulate the integrin-mediated cell spreading and the formation of focal adhesions possibly by binding to focal adhesion kinase (FAK) and the downregulation of FAK tyrosin phosphorylation (Tamura et al., 1999, 1998). In addition, binding of PTEN to Bazooka/Par3 (Von Stein et al., 2005), can result in its recruitment to actin rich regions and the establishment of apical membrane identity by dephosphorylating PIP3 into PIP2 (Shewan et al., 2011). Interestingly, Bazzoka/Par3 can recruit PTEN via its PDZ3 domain and thus could relocate PTEN to PIP3/PIP2-rich domains recognized by its PDZ2 domain (Wu et al., 2007). Drebrin (Developmentally regulated brain protein) is another recently identified interacting protein of PTEN, which binds to actin filaments (Kreis et al., 2013). Drebrin is known to change the property of the actin cytoskeleton to control the formation of filopodia (Hayashi et al., 1996), potentially involving CDK5 (Cyclin-dependent kinase 5) mediated phosphorylation at S142 (Worth et al., 2013). Phosphorylation at another Drebrin site, S647, is targeted by PTEN in a PI3K-independent fashion in response to signals that induce a dynamic remodeling of the cytoskeleton (Kreis et al., 2013). Therefore, it has become increasingly clear that not all cytoskeleton-dependent processes regulated by PTEN depend on the PIP3 lipid phosphatase activity.

As described in previous sections, PTEN is dynamically localized to specialized sub-cellular compartments and organelles. This dynamic recruitment implies a tight temporal and spatial regulation and current evidence highlights the contribution of PTEN protein modifications and formation of distinct protein-protein interactions in this process. Given the wide implication of PTEN in various pathological conditions, in the following sections, we attempt to summarize and emphasize the putative significance of compartmentalized PTEN pools in the progression of different neurodevelopmental diseases and neurodegenerative conditions.

## PTEN and neurodevelopmental diseases

### PTEN hamartoma tumor syndrome (PHTS)

The term, PTEN hamartoma tumor syndromes (PHTS), refers to a collection of clinically distinct syndromes molecularly defined by germline PTEN mutations. These include Cowden syndrome (CS), Bannayan-Riley-Ruvalcaba syndrome (BRRS), Proteus syndrome (PS), and Proteus-like syndrome (PSL) (Eng, 2003; Mester and Eng, 2013). CS is a multiple hamartoma syndrome with a high risk for benign and malignant tumors. BRRS is associated with macrocephaly, intestinal hamartomatous polyposis, lipomas, and pigmented macules of the glans penis. PS is a complex and highly variable disorder involving congenital malformations and hamartomatous overgrowth of multiple tissues. PSL, in contrast, appears relatively undefined. It refers to individuals with significant clinical features of PS who do not meet the diagnostic criteria for this syndrome. Importantly, up to 85% of individuals who meet the diagnostic criteria for CS and 65% of individuals with a clinical diagnosis of BRRS have detectable PTEN mutations (Eng, 2003). PTEN mutations appear also to be present in patients with PL and PLS, yet it has been suggested that patients with bona fide PS did not have PTEN mutations (Mester and Eng, 2013). Nevertheless, PI3K/PTEN/Akt/mTORC1 signaling seems to have a dominant role in the neurodevelopmental and overgrowth defects associated with this group of syndromes (Mester and Eng, 2013). Indeed, recent studies have reported the presence of activating mutations in Akt1 in PS (Lindhurst et al., 2011) and activating mutations in PIK3CA and Akt1 in a subset of CS (Orloff et al., 2013).

The majority of PTEN missense mutations found in PHTS occur in the phosphatase domain of PTEN (Eng, 2003; Mester and Eng, 2013) and most of these result in full or partial inactivation of PTEN's PIP3 activity (Eng, 2003; Rodríguez-Escudero et al., 2011). One particular mutation, G129E, originally identified in two independent CS kindreds, abolishes only the lipid phosphatase activity of PTEN, whilst the activity of the protein phosphatase is preserved (Myers et al., 1998). This characteristic has been instrumental in studying PIP3-dependent and -independent roles of PTEN (Raftopoulou et al., 2004; Tibarewal et al., 2012; Zhang et al., 2012a). Several PTEN mutations occur also in the C-terminal part of the protein (examples are F241S, P246L, K289E, R335L, V343E, L345V, F347L in the C2 domain). Some of these have been shown to reduce catalytic activity and membrane association (Rodríguez-Escudero et al., 2011) and/or have been shown to interfere with posttranslational modifications that are crucial for subcellular targeting of PTEN to the nucleus (Trotman et al., 2007). Phenotypes that can be associated with PTEN germline mutations also include Lhermitte-Duclos disease (LDD). Most, if not all, adult-onset LDD (dysplastic gangliocytoma of the cerebellum, a hamartomatous overgrowth known to be a feature of CS) can be attributed to mutations in PTEN, even in the absence of other clinical signs of CS/BRRS. However, germline PTEN mutations appear rare in individuals with childhood-onset LDD (Eng, 2003).

The PTEN-associated neurological deficits frequently observed in this group of syndromes are macrocephaly, developmental delay, and mental retardation. Ataxia, tremor, and epilepsy have been also reported (Lachlan et al., 2007; Conti et al., 2012; Mester and Eng, 2013; Pilarski et al., 2013). It should be noted, however, that no strong genotype–phenotype correlations are known in PHTS to date (Mester and Eng, 2013). Studies regarding the properties of mutated PTEN in PHTS are generally lacking, although some evidence suggests a correlation between PHTS and PTEN defect in a specific subcellular compartment. For example, the PTEN K289E mutation identified in CS disturbs the translocate of PTEN into the nuclei of patient tissue (Trotman et al., 2007). Another example relates to a mutation in the C2 domain, PTEN R335V which is also responsible for CS. This PTEN mutant enzyme retains normal levels of activity in cells (Rodríguez-Escudero et al., 2011), but its interaction with the lipid bilayer may be altered as suggested in a model of PTEN membrane association (Lumb and Sansom, 2013). Active research using molecular interaction simulation and modeling approaches, as well as functional screening of PTEN mutations in cell-based assays (Rodríguez-Escudero et al., 2011; Lumb and Sansom, 2013; Nguyen et al., 2013), will no doubt prove helpful in further clarifying the effects of PTEN mutations associated with PHTS.

### Autism spectrum disorders (ASD)

PTEN has been established as one of the genetic factors that play a major role in autism spectrum disorders (ASD) (Zhou and Parada, 2012). ASD serves as an umbrella term for autism and related conditions pertinent to social-communication deficits, and restricted and repetitive behavior diagnosed in early childhood. Autism is also associated with sensory and motor abnormalities, sleep disturbance, epilepsy, attention deficit/hyperactivity disorder (ADHD)-like hyperactivity, intellectual disability, and mood disorders such as anxiety and aggression (Won et al., 2013). Germline PTEN mutations have been identified in 5–10% of autism patients, following the initial report that approximately 20% of individuals with autism spectrum disorders and macrocephaly carry germline PTEN mutations (Butler et al., 2005). Macrocephaly is also prevalent in PTHS patients with PTEN mutations (Mester et al., 2011; Mester and Eng, 2013) and it is one of the most frequent neuroanatomical abnormalities (20%) observed in ASD individuals (Won et al., 2013).

Several PTEN mouse models have provided strong evidence for a causative role of PTEN dysfunction in almost all morphological, anatomical, synaptic, and behavioral manifestations relevant to ASD. Deletion of PTEN in neural progenitor cells and embryonic neural stem cells (Nestin-Cre:Pten^loxp/loxp^) results in increased neural stem cells proliferation, enlarged cell size, and brain enlargement (Groszer et al., 2001). Deletion of PTEN in GFAP (glial fibrillary acidic protein)-expressing neural progenitors, astrocytes, and a large subset of hippocampal neurons, cerebellar granule neurons, and cortical pyramidal neurons (Fraser et al., 2004, 2008) results in numerous structural abnormalities such as dramatic hypertrophy of nuclei, somata, axon, and dendritic caliber, enlarged presynaptic terminals, as well as absence or enlarged postsynaptic densities. Furthermore, the enlarged dendritic processes in Pten conditional knock out (cKO) mice showed a substantial increase in the density of spines and abnormal spine morphology (Fraser et al., 2008). Mice also developed progressive macrocephaly, seizures, and ataxia (Backman et al., 2001; Kwon et al., 2001). Although an increase in spine density was also observed following PTEN shRNA (short-hairpin RNA) treatments, there seems to be subtle difference when compared to genetically induced PTEN-loss (Luikart et al., 2011). In the basolateral amygdala and the dentate gyrus PTEN shRNA treatment resulted in increased mushroom spine density and spine size, with correspondingly decreased thin protrusion density at more distal segments (Haws et al., 2014). These results suggest that rather than inducing de novo spinogenesis *per se*, PTEN deletion may interfere with normal morphological and functional maturation of spines (Haws et al., 2014). Nevertheless, the most informative animal model related to ASD is the NSE-Cre:Pten^loxp/loxp^ mouse with postnatal deletion of PTEN in selected neuronal populations (Dentate gyrus granule cells, CA3 hippocampal neurons, and selected populations of cortical neurons). This PTEN cKO model recapitulates many of the late-onset morphological and behavioral abnormalities associated with autistic symptoms as these mice exhibit macrocephaly, neuronal hypertrophy, and deficits in hippocampus-based social and cognitive behavior, hypersensitivity to sensory stimuli, anxiety, and epileptic seizures (Kwon et al., 2006). Notably, autism-relevant behaviors are detectable at a time when morphological abnormalities seem relatively subtle (Kwon et al., 2006). Subsequent studies using a tamoxifen-inducible Nestin-Cre:Pten^loxp/loxp^ mouse model, in order to delete PTEN specifically in postnatal neural stem cells in the subgranular zone of hippocampal dentate gyrus, recapitulated the general overgrowth phenotype seen in other models and some, but not all of the impairments associated with autism (Amiri et al., 2012).

PTEN's role in regulating neuron morphology, gross anatomical changes and behavior is largely dependent on its lipid phosphatase activity. As such, in all PTEN KO and knockdown models studied to date, the major biochemical result of PTEN deletion invariably observed is an enhancement in Akt/mTORC1 signaling pathway activity. This is consistent with the primary defect of PTEN loss being an increase in PIP3 production and PI3K signaling. However, pharmacological inhibition of mTORC1 or co-deletion of PDK1 has been shown to reverse most—but not always all—phenotypes associated with PTEN deficiency *in vivo* (Zhou et al., 2009; Sperow et al., 2012). Although this could be due to subtle or specific requirements of PTEN's lipid phosphatase activity depending on the cellular or developmental context, it is also suggestive of, perhaps latent, PI3K-independent functional outputs of PTEN which are important for proper brain development.

#### ASD-associated PTEN mutations

The study of the mutational spectrum of PTEN in ASD patients has revealed at least 25 mutations to date, of which 16 are amino-acid substitutions (Table 1) (Rodríguez-Escudero et al., 2011; Klein et al., 2013; Hobert et al., 2014). More than half of these mutations have also been identified in PHTS patients or as somatic mutations in sporadic cancers. They are scattered along the PTEN phosphatase and the C2 domain, and are absent from the N-terminal and the C-terminal regions. The exact effect of ASD-unique mutations on the properties of PTEN (i.e., residual phosphatase activity, altered membrane/protein interactions, protein stability) is still poorly characterized. A number of recent studies have suggested the possibility that ASD mutations are less severe (i.e., mutant forms retain partial activity) when compared to tumor-related mutations. The mostly studied ASD mutation in the context of its effect on PTEN activity is PTEN H93R. This mutation also identified in CS, appears to result in an 85% decrease in PTEN's PIP3 phosphatase activity when measured against water-soluble PIP3 *in vitro*. Yet this mutation increased PTEN association with PS-rich liposomes *in vitro*, as well as increased membrane localization *in vivo* (Redfern et al., 2010). Notably, when compared to wild-type PTEN, PTEN H93R was unable to fully reduce PI3K-dependent Akt phosphorylation in U87MG glioblastoma cells, indicating a partial loss of activity *in vivo* (Redfern et al., 2010). Also, using a heterologous yeast PI3K-activity reconstitution system, PTEN H93R appeared to retain residual phosphatase activity when compared to PHTS PTEN mutants (Rodríguez-Escudero et al., 2011). Thus, it would appear that complete loss of PTEN phosphatase activity and/or PTEN stability is rather an infrequent event in ASD cases (Zhou and Parada, 2012). However, in addition to understanding the molecular effect of these ASD-unique mutations, their exact effect on the modulation of morphology and synaptic plasticity by PTEN awaits detailed analyses in neuronal cells.

**Table 1:** **ASD-associated missense mutations**.

**Mutation^a^**	**Macrocephaly^b^**	**Cancer^c^**	**Domain**	**Activity^d^**	**Notes^e^**
M1I	+				
P38H	+		Phosphatase		
Y68N	+	+	Phosphatase		Y68D in PS
L70V	+		Phosphatase		
H93R	+	+	Phosphatase	++	CS
H118P	+		Phosphatase	++	
H123Q	+		Phosphatase	–	H123D in CS, P-loop
R130L	+	+	Phosphatase		Frequently mutated in cancer, P-loop
E157G	+		Phosphatase	++	
R173H	+	+	Phosphatase	++	CS, phosphatase-C2 interface
Y176C	+		Phosphatase	+++	CS, phosphatase-C2 interface
F241S	+	+	C2	– /+	CS
V255A	+	+	C2		
D252G	+	+	C2	+++	Phosphatase-C2 interface
N276S	+		C2	+++	Phosphatase-C2 interface
D326N	+		C2		

a *Data from Rodríguez-Escudero et al. (2011), Hobert et al. (2014), Klein et al. (2013), Butler et al. (2005), Orrico et al. (2009), McBride et al. (2010), Varga et al. (2009), Buxbaum et al. (2007). Truncations R130X, L139X, Y178X, R335X, and R355X that presumably correspond to unstable/non-functional proteins are not included in the table*.

b *Association with macrocephaly*.

c *Occurrence as somatic mutation in cancers (COSMIC database)*.

d *Activity in the yeast system (Rodríguez-Escudero et al., 2011 – means no activity, +++ means full activity compared to wild type PTEN)*.

e *Association with PHTS is indicated (PS, Proteus Syndrome; CS, Cowden Syndrome), as well as the catalytic or structural significance*.

#### PTEN functions in dendritic spines

Given the dominant effects of PTEN depletion on neuronal cell morphology and gross architecture of brain structures, it has been important to delineate the exact PTEN requirements for specific synaptic, behavioral and neuroanatomical defects observed in ASD-related PTEN mouse models. Importantly, a series of recent studies have highlighted that PTEN deletion results in specific early-onset deficits in postsynaptic plasticity that may even precede minor and gross anatomical changes. Depending on the experimental approach, the developmental stage and the mode of PTEN inactivation, loss of PTEN has been shown to result in changes in synaptic function including both forms of synaptic plasticity, long-term potentiation (LTP) and long-term depression (LTD) (Sperow et al., 2012; Takeuchi et al., 2013). Often, these changes are observed in the absence of the hypertrophy phenotypes that are characteristic of PTEN loss in postmitotic neurons (i.e., increases in soma size and dendritic spine density and dendrite arborization) (Sperow et al., 2012), suggesting that a primary role of PTEN is the regulation of synaptic function within the postsynaptic compartment. Thus, it is tempting to speculate that there is a spine-specific PTEN pool, which actively participates in the regulation of essential synaptic functions locally.

It has been suggested that a continuous turnover of PIP3 at the spine may support synaptic function, at least by maintaining synaptic retention of AMPAR (α-Amino-3-hydroxy-5-methyl-4-isoxazolepropionic acid receptor) under basal conditions (Arendt et al., 2010). Furthermore, PI3K is activated upon LTP induction and it is also required for various forms of LTD (Kim et al., 2011a). PTEN is recruited to the PSD upon NMDAR activation, primarily via PDZ-dependent interactions with PSD-95 (Jurado et al., 2010). In this case, it has been proposed that PTEN lipid phosphatase activity is able to drive depression of AMPAR-mediated synaptic responses. This activity is specifically required for NMDA receptor-dependent LTD (Jurado et al., 2010). Other studies have suggested that the recruitment of PTEN into the postsynaptic compartment may lead to modifications in proteins implicated in synaptic function involving specifically the protein phosphatase activity (Kreis et al., 2013). PTEN's protein phosphatase activity in neurons has been less well-characterized, although increasing evidence suggests that it may be important for regulating PTEN itself through auto-phosphorylation as well as through other protein substrates. For example, overexpression of a protein phosphatase-deficient PTEN mutant, PTEN Y138L (Tibarewal et al., 2012), in organotypic hippocampal slice cultures does not phenocopy the effect of wild-type PTEN or PTEN G129E in reducing spine density (Zhang et al., 2012a). It has been suggested that autodephosphorylation of PTEN in this context may function in de-repressing the exposure of the C-terminal PDZ-binding domain, thus allowing for PDZ domain-PTEN interactions to take place (Zhang et al., 2012a). Alternatively, PTEN's protein phosphatase activity could be directed to other protein substrates located in dendritic spines, such as Drebrin (Kreis et al., 2013). Drebrin phosphorylation at S647 is under the control of PTEN and it depends on direct complex formation of the two proteins and synaptic activity (Kreis et al., 2013). In conclusion, increasing the understanding of the molecular mechanisms that controls PTEN movement into and out of the postsynaptic compartment, and those that direct PTEN lipid/protein phosphatase activity might prove to be key to fully comprehend the impact of PTEN mutations associated with neurological disorders.

## PTEN and axon regeneration

PTEN's role in regeneration became evident in a seminal study that induced genetic deletion of PTEN, specifically in adult retinal ganglion cells, which promoted regeneration of axonal fibers after subjection to an optic nerve crush injury (Park et al., 2008). Since then, research in finding ways to modify the expression or the activity of PTEN have become of great interest in this field. This might be particularly important for CNS neurons since axonal regeneration, contrary to neurons of the PNS (peripheral nervous system), is extremely limited following injury, which is due to both an inhibitory environment present at the side of injury as well as a general diminished regenerative capacity of affected neurons. Subsequent studies have shown that upon nerve transection of PNS neurons, axon outgrowth is enhanced following local inhibition of PTEN using the pharmacological PTEN inhibitor BpV(pic) or following siRNA knockdown of PTEN (Christie et al., 2010). PTEN loss using genetic deletion or shRNA approaches has also been shown to increase corticospinal tract sprouting, axon regeneration, and the enhanced formation of synaptic structures in models for spinal cord injury using unilateral pyramidotomy, dorsal hemisection, or complete spinal cord crush (Liu et al., 2010; Zukor et al., 2013).

For successful regeneration after an injury to occur, the injured axon tip must be remodeled to reform a growth cone. This transformation involves major changes in the cytoskeleton, as well as changes in membranous and cytoplasmic components that are necessary for the synthesis of new molecules. Several downstream effectors of PTEN have been shown to convey the information of axon regrowth. The PI3K/Akt/mTORC1 pathway, in particular, which regulates protein synthesis, is one of the important targets involved in axon regrowth. For example, pyramidotomy induces the loss of mTORC1 activity, an event that can be prevented by deletion of PTEN (Liu et al., 2010). Inhibition of mTORC1 using rapamycin largely neutralized the regenerative capacity of PTEN deleted neurons following axotomy (Park et al., 2008), suggesting that the injured axon tip benefits from increased mTORC1 activity in terms of the growth potential and regenerative growth. However, significant axon regeneration was still observed when inhibiting mTORC1, suggesting that other pathways may be involved (Park et al., 2008). Another study also suggested mTORC1-independent pathways, where the increased neurite outgrowth after PTEN deletion in the PNS, was not affected by rapamycin (Christie et al., 2010). Interestingly, combined activation of both the JAK/Stat and the PI3K/Akt/mTORC1 further increases the regenerative capacity of CNS neurons in the optic nerve crush model (Sun et al., 2011), demonstrating the significant interplay between different signaling pathways in promoting regenerative growth as well as neuronal survival (Luo and Park, 2012). Upstream of PTEN, signals mediated by inhibitors of axon growth such as MAGs or Sema3A could be responsible for preventing axon regeneration by activating PTEN. Indeed, deletion of PTEN resulted in a significant, although partial rescue of neurite outgrowth on MAG expressing CHO cells (Perdigoto et al., 2011).

The prospect to increase the regenerative capacity of damaged neurons by inhibiting PTEN has been of great interest for therapeutic strategies. In a mouse model for spinal muscular atrophy (SMA), the knock down of PTEN rescued disease associated defects in axon length, increased survival and restored growth cone sizes (Ning et al., 2010). Similarly, inhibition of PTEN using BpV(pic) has been shown to have a beneficial effect on spinal cord injuries, leading to increases in the number of motoneurons at the injury epicenter and improving forelimb articulation (Walker et al., 2012). Inhibition of PTEN may also be a promising target in amyotrophic lateral sclerosis (ALS), a well-studied degenerative motor neuron disease that that can be linked to inactivating mutations of the Cu/Zn superoxide dismutase SOD1. Using microarray analysis of SOD1 deficient ALS spinal cord motor neurons, several components of the PI3K signaling pathway were identified to be dysregulated, which included PTEN (Kirby et al., 2011).

Collectively, these studies suggest that, at physiological settings, PTEN functions in restricting regenerative growth in the PNS and CNS neurons, and this is in agreement with PTEN's role in inhibiting the growth-promoting PI3K/Akt/mTORC1 pathway. Furthermore, it appears that deletion or inhibition of PTEN offers advanced neuroprotection by increasing neuronal survival. One of the key questions will entail the analyses of the minimal pool of PTEN that—once it is lost—induces regenerative growth response. Thus, unraveling the specific functions of PTEN in the different subcellular compartments of neurons should bring new insights into the mechanisms necessary for the establishment of a robust and sustained regeneration.

## PTEN and neurodegenerative conditions

### PTEN and Alzheimer's disease

AD involves the degeneration of neurons and the accumulation of pathological highly phosphorylated Tau protein species, which eventually generate neurofibrillary tangles. The driving force for AD, however, involves the overproduction of the amyloid β -peptides that form the extracellular deposits found in the brains of patients with AD, the amyloid plaques. It was initially shown that stimulation of the PI3K/Akt signaling pathway *in vitro* through insulin-like growth factor may protect Aβ induced neurotoxicity (Doré et al., 1997; Wei et al., 2002). This was in line with the fact that GSK-3, which operates downstream of the PI3K signaling pathway, had been identified as a potential kinase responsible for causing hyper-phosphorylation of Tau (Hanger et al., 1992). Later, the function of PI3K in neuronal survival and AD showed the requirement of a more defined coordination of the different components of the pathway. Contrary to initial expectations, an overactivation of PI3K signaling was reported in postmortem brain tissue in both immunohistochemistry and immunobiochemistry with an increase in Akt activation, a loss of neuronal cytosolic Akt and a loss and modified localization of PTEN in the temporal cortex and hippocampus of AD patients (Griffin et al., 2005). Interestingly, a loss of nuclear PTEN was observed in neurons of the CA1, subiculum, and entorhinal cortex, as well as in the temporal cortex of AD cases (Griffin et al., 2005). In agreement with this, another study showed that PTEN re-distributed from the nucleus to the cytoplasm in regions such as the hippocampus and the entorhinal cortex of AD tissue and accumulated in intracellular neurofibrillary tangles (Sonoda et al., 2010). It has been suggested that the overactivation of the PI3K signaling pathway and the observed decreases in PTEN protein levels originate from a pro-survival response aimed at compensating for the disease. Decreased PTEN protein abundances may also impact on the phosphorylation state of Tau. Indeed, the lipid phosphatase activity of PTEN may affect Tau phosphorylation as *in vitro* studies have shown that PTEN can affect the formation of Tau aggregates in a GSK3-independent manner (Kerr et al., 2006; Zhang et al., 2006). The exact function of PTEN in Alzheimer disease remains, however, poorly understood, due to the complexity of the disease and the existence of numerous compensatory feedback loops within the PI3K signaling pathway. Decisive proof of whether the observed loss of PTEN in the nucleus in AD tissue leads to an apoptotic or a prosurvival signal remains elusive. Similarly, detailed information on the function of PTEN and/or its modified localization in other subcellular compartments such as the ER, the mitochondria or the MAMs are still not available. Nevertheless, PTEN may play a key role in the regulation of calcium signaling and apoptotic responses that are both perturbed in AD and are associated with these compartments. For instance, as mentioned in the previous section, PTEN recruited at the MAM-ER membranes (Bononi et al., 2013) could have an effect on modification in the calcium transfer from the ER to the mitochondria that has been observed following Aβ treatment (Hedskog et al., 2013). A similar contribution of PTEN could potentially relate to its interaction with the pro-apoptotic protein Bax at the mitochondria and regulation of apoptotic signaling pathways (Zu et al., 2011). Again, these ideas, though plausible, have not yet been tested in the context of AD research.

### PTEN and parkinson's disease

Parkinson's disease (PD) is the second most prevalent neurodegenerative disorder after Alzheimer's. It is characterized by progressive loss of dopaminergic neurons in the substantia nigra pars compacta. Earlier studies provided links for PTEN's involvement in the pathogenesis of PD by virtue of its indirect and direct interactions, respectively, with two prominent PD-associated genes, PINK1 and DJ-1. However, more recent studies utilizing primarily neuron subtype-specific PTEN cKO mice have suggested diverse roles for PTEN and the Akt/mTOR pathway in dopaminergic neurons. It has been shown, for instance, that inhibition of PTEN in dopaminergic cell lines significantly inhibits the neuronal death caused by 1-methyl-4-phenylpyridinium (MPP+), an established *in vitro* model of PD toxicity (Zhu et al., 2007). Dopaminergic neuron-specific deletion of PTEN recapitulated the hypertrophy phenotype observed in other cKO mouse models (Diaz-Ruiz et al., 2009). In addition, PTEN inactivation protected dopaminergic neurons and significantly enhanced dopamine-dependent behavioral functions in cKO mice after a progressive 6-hydroxydopamine (6OHDA) lesion, where application of this neurotoxin into the striatum results in progressive loss of neurons in the ventral mesencephalon (Diaz-Ruiz et al., 2009). Subsequent studies in adult mice using an inducible Cre system showed that specific ablation of PTEN in adult dopaminergic neurons is neuroprotective in genetic and neurotoxin-induced mouse models of PD (Domanskyi et al., 2011). In this case, PTEN deletion attenuated the loss of tyrosine hydroxylase-positive cells after 6OHDA treatment. Also, specific ablation of an essential factor controlling ribosomal RNA transcription, TifIa, in adult mouse dopaminergic neurons represses mTOR signaling and leads to progressive neurodegeneration and PD-like phenotype. Here, PTEN deletion rescued locomotor impairments caused by absence of Tifla (Domanskyi et al., 2011). These more recent studies are in accordance with an earlier report showing that overactivation of PI3K signaling, by adenoviral transduction of a membrane-localized Akt1 form into substantia nigra neurons, offers protection against 6OHDA (Ries et al., 2006). Another example of the general applicability and beneficial effects of PTEN deletion in PD mouse models is the enhanced survival, function and integration of grafted PTEN^−/−^ dopaminergic neurons within the striatum of MitoPark mice, a model that shows progressive parkinsonism after specific inactivation of the mitochondrial respiratory chain in midbrain dopaminergic neurons (Zhang et al., 2012b).

A specific requirement for PTEN in the regulation of axon terminal morphology of dopaminergic neurons has been uncovered in neurons deficient in an essential macroautophagy component, Atg7 (Inoue et al., 2013). In these mice, midbrain dopaminergic neurons exhibit strikingly enlarged axon terminals and also late-onset degeneration *in vivo*. Interestingly, although PTEN-deficient dopaminergic neurons show no alteration at the axon terminal, concomitant deletion of PTEN and Atg7 results in a exacerbation of the axon terminal size seen in Atg7 deficient neurons in the absence of any degeneration phenotype (Inoue et al., 2013). These results suggest that macroautophagic activity, at least in dopaminergic neurons, may limit the impact of PTEN/PI3K/mTOR pathway on axon terminal morphology (Inoue et al., 2013).

In conclusion, an overall protective effect against neuronal death has been demonstrated when PTEN is deleted or inactivated in dopaminergic neurons, which is likely to reflect a generalized response due to activation of the PI3K/Akt/mTOR signaling pathway.

## Concluding remarks

Numerous specific modes of PTEN functions have been identified that are restricted to diverse subcellular compartments and involved in mediating a plethora of cellular responses. It has become increasingly clear that PTEN functions are not exclusively restricted to targeting phosphoinositides in membranous compartments; instead, PTEN specific cellular responses also involve its protein phosphatase activity or, no phosphatase activity at all. On one hand, PTEN inhibition has become a potentially attractive therapeutic intervention in certain settings. Yet, on the other hand, PTEN activation could be beneficial in other conditions. So far, chemical compounds that can act as PTEN inhibitors have been shown to substantially corroborate findings from studies utilizing deletion and silencing approaches. In principal, rational design of chemical compounds or peptides to shift, or prevent PTEN's localization to a specific subcellular compartment, to alter binding to a prominent protein partner, or to impose specific structural conformations on PTEN are plausible. Furthermore, the implications of using PTEN itself as an exogenous factor are only now beginning to be appreciated. In this review, we have attempted to detail some of the roles of PTEN that are, or may turn out to be involved, in mediating cellular responses in the developing, the mature, as well as the diseased neuron. No doubt, the interplay between the mechanisms that coordinate subcellular targeting in the context of controlling enzymatic activity will require detailed attention and have to be further explored.

### Conflict of interest statement

The authors declare that the research was conducted in the absence of any commercial or financial relationships that could be construed as a potential conflict of interest.

## Abbreviations

ASD: Autism spectrum disorders
AMPAR: α-Amino-3-hydroxy-5-methyl-4-isoxazolepropionic acid receptor
BRRS: Bannayan-Riley-Ruvalcaba syndrome
cKO: conditional knockout
CNS: central nervous system
CS: Cowden syndrome
Drebrin: Developmentally regulated brain protein
DRG: dorsal root ganglia
FRET: Fluorescence resonance energy transfer
GAP: GTPase Activating Protein
GEF: Guanine nucleotide Exchange Factor
GFAP: glial fibrillary acidic protein
GPCR: G-protein coupled receptor
GSK3: glycogen synthase kinase
InsP3R: inositol 1,4,5-trisphosphate receptor
LDD: Lhermitte-Duclos disease
LTD: long-term depression
LTP: long-term potentiation
MAGs: myelin-associated glycoproteins
mTOR: mammalian target of rapamycin
MAM: mitochondria-associated membrane
Nedd4: neural precursor cell-expressed developmentally downregulated gene 4
Ndfip1: Nedd4 family interacting protein-1
NEP: Neutral Endopeptidase
NGF: nerve growth factor
NHERF: Na(+)/H(+) exchanger regulatory factor
NMDAR: N-methyl-D-aspartate receptor
NSE: neuron-specific enolase
PBM: PIP2-binding motif
PDGFR: Platelet-derived growth factor receptor
PDK1: phosphoinositide-dependent kinase 1
PDZ: post synaptic density protein (PSD95), Disc large tumor suppressor (Dlg1), and zonula occludens-1 protein (zo-1)
PH: pleckstrin homology
PHLPP: PH domain and Leucine-rich repeat Protein Phosphatase
PHTS: PTEN hamartoma tumor syndromes
PI3,4P2: phosphatidylinositol 3,4-bisphosphate
PI3K: phosphoinositide 3 kinase
PIP2: phosphatidylinositol 4,5-bisphosphate
PIP3: phosphatidylinositol 3,4,5-trisphosphate
PNS: peripheral nervous system
PS: Proteus syndrome
PSD: post synaptic density
PSL: Proteus-like syndrome
PTEN: Phosphatase and tensin homolog located on chromosome 10
RHEB: Ras Homolog Enriched in Brain
SHIP: SH2-domain containing inositol 5-phosphatase
shRNA: short-hairpin RNA
TBI: traumatic brain injury
TSC: Tuberous Sclerosis Complex.
